# Crystal structure of 1-((1*E*)-{(*E*)-2-[(2-hydroxy­naphthalen-1-yl)methyl­idene]hydrazin-1-yl­idene}meth­yl)naphthalen-2-ol

**DOI:** 10.1107/S205698901500972X

**Published:** 2015-05-28

**Authors:** Paranthaman Vijayan, Periasamy Viswanathamurthi, Michel Fleck, Sugumar Paramasivam, Ponnuswamy Mondikalipudur Nanjappagounder

**Affiliations:** a Department of Chemistry, Periyar University, Salem 636 011, India; b Institute of Mineralogy and Crystallography Geozentrum, University of Vienna, Althanstrasse 14, A-1090 Vienna, Austria; c Centre of Advanced Studies in Crystallography and Biophysics, University of Madras, Guindy Campus, Chennai 600 025, India

**Keywords:** crystal structure, Schiff base derivative, intra­molecular hydrogen bonding

## Abstract

The complete mol­ecule of the title compound, C_22_H_16_N_2_O_2_, is generated by a crystallographic inversion centre at the mid-point of the central N—N bond. Two intra­molecular O—H⋯N hydrogen bonds occur.

## Related literature   

For general background to Schiff base derivatives, see: Hoshino (1998 ▸); Kalaivani *et al.* (2013 ▸); Vijayan *et al.* (2014 ▸).
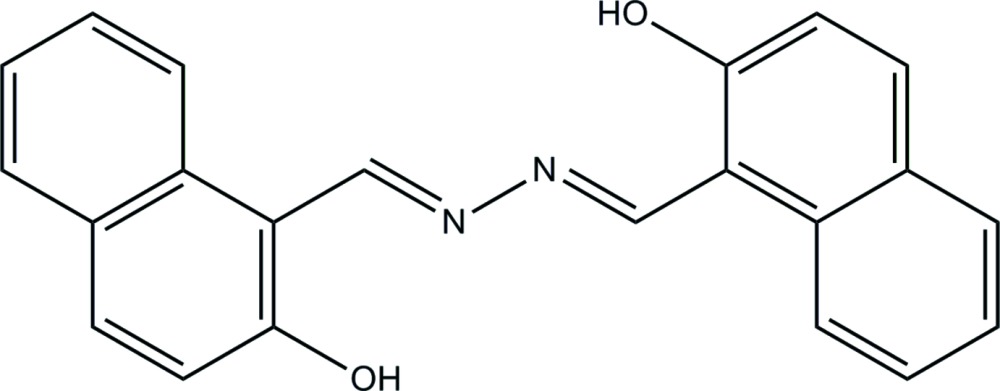


## Experimental   

### Crystal data  

C_22_H_16_N_2_O_2_
*M* *_r_* = 340.37
Monoclinic, 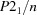

*a* = 8.5680 (7) Å
*b* = 6.1020 (5) Å
*c* = 15.9870 (6) Å
β = 91.191 (5)°
*V* = 835.65 (10) Å^3^
*Z* = 2
Mo *K*α radiation
μ = 0.09 mm^−1^
*T* = 293 K
0.22 × 0.20 × 0.18 mm

### Data collection  

Bruker SMART APEXII CCD diffractometer
Absorption correction: multi-scan (*SADABS*; Bruker, 2008 ▸) *T*_min_ = 0.981, *T*_max_ = 0.984
1907 measured reflections
1907 independent reflections
1859 reflections with *I* > 2σ(*I*)

### Refinement  

*R*[*F* ^2^ > 2σ(*F* ^2^)] = 0.007
*wR*(*F* ^2^) = 0.019
*S* = 1.03
1907 reflections
122 parameters
1 restraint
H atoms treated by a mixture of independent and constrained refinement
Δρ_max_ = 0.02 e Å^−3^
Δρ_min_ = −0.08 e Å^−3^

### 

Data collection: *APEX2* (Bruker, 2008 ▸); cell refinement: *SAINT* (Bruker, 2008 ▸); data reduction: *SAINT*; program(s) used to solve structure: *SHELXS97* (Sheldrick, 2008 ▸); program(s) used to refine structure: *SHELXL97* (Sheldrick, 2008 ▸); molecular graphics: *ORTEP-3 for Windows* (Farrugia, 2012 ▸); software used to prepare material for publication: *SHELXL97* and *PLATON* (Spek, 2009 ▸).

## Supplementary Material

Crystal structure: contains datablock(s) global, I. DOI: 10.1107/S205698901500972X/hb7408sup1.cif

Structure factors: contains datablock(s) I. DOI: 10.1107/S205698901500972X/hb7408Isup2.hkl

Supporting information file. DOI: 10.1107/S205698901500972X/hb7408Isup3.cml

**Fig. 1.**
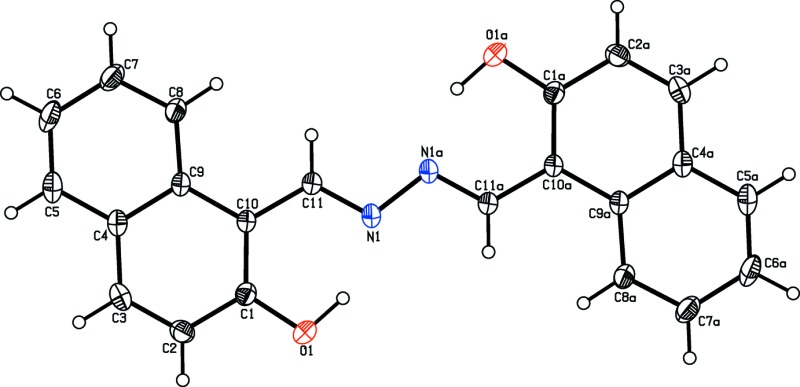
The molecular structure of the title compound, showing displacement ellipsoids drawn at 50% probability level.

. DOI: 10.1107/S205698901500972X/hb7408fig1.tif
The mol­ecular structure of the title compound, showing displacement ellipsoids drawn at 50% probability level.

CCDC reference: 1401958

Additional supporting information:  crystallographic information; 3D view; checkCIF report

## Figures and Tables

**Table 1:** Hydrogen-bond geometry (, )

*D*H*A*	*D*H	H*A*	*D* *A*	*D*H*A*
O1H1N1	0.98	1.67(1)	2.5671(3)	151(1)

## supplementary crystallographic information

### S1. Comment

Schiff bases are important ligands, as moderate electron donors with a chelating structure and control the behaviour of metal ions in a diverse range of applications (Hoshino, 1998). Dithiocarbazate compounds are an important class of Schiff bases which can be easily obtained by condensation of dithiocarbazides with aldehydes or ketones. In particular hydrazone containing naphthalene ring compounds have drawn much attention because of their biological activities such as DNA/BSA binding affinities and anticancer activities *in vitro* (Kalaivani *et al.*, 2013; Vijayan *et al.*, 2014).

The *ORTEP* plot of the molecule is shown in Fig.1. The title compound (I), crystallized in the monoclinic spacegroup *P*2_1_/*n* with half molecule in the asymmetric unit. Pair of molecules related by an crystallographic inversion centre generate another half of the molecule.

### S2. Experimental

The title compound was obtained by the reaction of *S*-benzyldithiocarbazate and 2-Hydroxy-1-napthaldehyde in boiling ethanol. The unexpected formation of the hydrazone was probably due to the decomposition of *S*-benzyldithiocarbazate in solution resulting in the formation of hydrazine, which then reacted with 2-hydroxy-1-napthaldehyde to form the corresponding hydrazone. The dithiocarbazates are known to decompose on heating.

### S3. Refinement

All non-hydrogen atoms were refined anisotropically. Hydrogen atoms were located geometrically (aromatic C—H 0.95 Å, secondary alkane C—H 0.99 Å, tertiary alkane C—H 1.0 Å) and refined using a riding model with the isotropic displacement parameters fixed at *U_iso_*= 1.2 times *U_eq_* of the parent carbon for all of the hydrogen atoms.

### Crystal data

**Table:**

C_22_H_16_N_2_O_2_	*F*(000) = 356
*M**_r_* = 340.37	*D*_x_ = 1.353 Mg m^−^^3^
Monoclinic, *P*2_1_/*n*	Mo *K*α radiation, λ = 0.71073 Å
Hall symbol: -P 2yn	Cell parameters from 1859 reflections
*a* = 8.5680 (7) Å	θ = 2.6–27.5°
*b* = 6.1020 (5) Å	µ = 0.09 mm^−^^1^
*c* = 15.9870 (6) Å	*T* = 293 K
β = 91.191 (5)°	Block, yellow
*V* = 835.65 (10) Å^3^	0.22 × 0.20 × 0.18 mm
*Z* = 2	

### Data collection

**Table:**

Bruker SMART APEXII CCD diffractometer	1907 independent reflections
Radiation source: fine-focus sealed tube	1859 reflections with *I* > 2σ(*I*)
Graphite monochromator	*R*_int_ = 0.0000
ω and φ scans	θ_max_ = 27.5°, θ_min_ = 2.6°
Absorption correction: multi-scan (*SADABS*; Bruker, 2008)	*h* = −11→11
*T*_min_ = 0.981, *T*_max_ = 0.984	*k* = 0→7
1907 measured reflections	*l* = 0→20

### Refinement

**Table:**

Refinement on *F*^2^	Primary atom site location: structure-invariant direct methods
Least-squares matrix: full	Secondary atom site location: difference Fourier map
*R*[*F*^2^ > 2σ(*F*^2^)] = 0.007	Hydrogen site location: inferred from neighbouring sites
*wR*(*F*^2^) = 0.019	H atoms treated by a mixture of independent and constrained refinement
*S* = 1.03	*w* = 1/[σ^2^(*F*_o_^2^) + (0.008*P*)^2^where *P* = (*F*_o_^2^ + 2*F*_c_^2^)/3
1907 reflections	(Δ/σ)_max_ = 0.001
122 parameters	Δρ_max_ = 0.02 e Å^−^^3^
1 restraint	Δρ_min_ = −0.08 e Å^−^^3^

### Special details

**Table:**

Geometry. All e.s.d.'s (except the e.s.d. in the dihedral angle between two l.s. planes) are estimated using the full covariance matrix. The cell e.s.d.'s are taken into account individually in the estimation of e.s.d.'s in distances, angles and torsion angles; correlations between e.s.d.'s in cell parameters are only used when they are defined by crystal symmetry. An approximate (isotropic) treatment of cell e.s.d.'s is used for estimating e.s.d.'s involving l.s. planes.
Refinement. Refinement of *F*^2^ against ALL reflections. The weighted *R*-factor *wR* and goodness of fit *S* are based on *F*^2^, conventional *R*-factors *R* are based on *F*, with *F* set to zero for negative *F*^2^. The threshold expression of *F*^2^ > σ(*F*^2^) is used only for calculating *R*-factors(gt) *etc*. and is not relevant to the choice of reflections for refinement. *R*-factors based on *F*^2^ are statistically about twice as large as those based on *F*, and *R*- factors based on ALL data will be even larger.

### Fractional atomic coordinates and isotropic or equivalent isotropic displacement parameters (Å^2^)

**Table:**

	*x*	*y*	*z*	*U*_iso_*/*U*_eq_	
C1	0.37033 (2)	0.02490 (3)	0.395255 (11)	0.03120 (4)	
C2	0.32482 (2)	−0.16485 (3)	0.350605 (13)	0.03653 (5)	
H2	0.2610	−0.2683	0.3755	0.044*	
C3	0.37391 (2)	−0.19647 (3)	0.271551 (13)	0.03672 (5)	
H3	0.3450	−0.3240	0.2434	0.044*	
C4	0.46786 (2)	−0.04107 (3)	0.230675 (12)	0.03129 (5)	
C5	0.51123 (3)	−0.06977 (4)	0.146509 (13)	0.04195 (5)	
H5	0.4815	−0.1967	0.1182	0.050*	
C6	0.59566 (3)	0.08510 (5)	0.106276 (13)	0.04842 (6)	
H6	0.6213	0.0654	0.0505	0.058*	
C7	0.64395 (3)	0.27462 (5)	0.149184 (13)	0.04576 (6)	
H7	0.7021	0.3802	0.1217	0.055*	
C8	0.60616 (2)	0.30547 (4)	0.231137 (12)	0.03661 (5)	
H8	0.6408	0.4310	0.2588	0.044*	
C9	0.51573 (2)	0.15099 (3)	0.274652 (11)	0.02726 (4)	
C10	0.46705 (2)	0.18089 (3)	0.359484 (10)	0.02658 (4)	
C11	0.51614 (2)	0.37178 (3)	0.406697 (11)	0.02991 (4)	
H11	0.5827	0.4727	0.3824	0.036*	
N1	0.46961 (2)	0.40510 (3)	0.481933 (10)	0.03414 (4)	
O1	0.31470 (2)	0.04548 (3)	0.472921 (9)	0.04471 (5)	
H1	0.3596 (5)	0.1824 (7)	0.4943 (2)	0.0995 (13)*	

### Atomic displacement parameters (Å^2^)

**Table:**

	*U*^11^	*U*^22^	*U*^33^	*U*^12^	*U*^13^	*U*^23^
C1	0.03470 (9)	0.03336 (10)	0.02553 (9)	0.00099 (8)	0.00052 (7)	−0.00058 (7)
C2	0.03940 (11)	0.03200 (10)	0.03805 (11)	−0.00574 (8)	−0.00259 (8)	0.00167 (8)
C3	0.03929 (11)	0.03106 (10)	0.03943 (11)	0.00132 (8)	−0.00822 (8)	−0.00859 (8)
C4	0.02942 (9)	0.03540 (10)	0.02889 (9)	0.00595 (8)	−0.00317 (7)	−0.00776 (8)
C5	0.04097 (11)	0.05126 (13)	0.03346 (11)	0.00604 (10)	−0.00287 (8)	−0.01694 (9)
C6	0.04610 (12)	0.07303 (17)	0.02636 (10)	0.00235 (12)	0.00672 (9)	−0.01219 (10)
C7	0.04615 (12)	0.06073 (15)	0.03086 (10)	−0.00308 (11)	0.01184 (9)	−0.00236 (10)
C8	0.03867 (10)	0.04315 (12)	0.02823 (10)	−0.00353 (9)	0.00582 (8)	−0.00574 (8)
C9	0.02591 (8)	0.03176 (9)	0.02405 (8)	0.00337 (7)	−0.00108 (6)	−0.00519 (7)
C10	0.02861 (9)	0.02830 (9)	0.02281 (8)	0.00107 (7)	−0.00030 (6)	−0.00319 (7)
C11	0.03341 (9)	0.03091 (10)	0.02545 (9)	−0.00130 (7)	0.00150 (7)	−0.00289 (7)
N1	0.04353 (9)	0.03257 (9)	0.02637 (8)	−0.00328 (7)	0.00204 (7)	−0.00689 (7)
O1	0.05713 (10)	0.04698 (10)	0.03052 (8)	−0.01041 (8)	0.01283 (7)	−0.00151 (7)

### Geometric parameters (Å, º)

**Table:**

C1—O1	1.3451 (2)	C6—H6	0.9300
C1—C10	1.3927 (3)	C7—C8	1.3691 (3)
C1—C2	1.4109 (3)	C7—H7	0.9300
C2—C3	1.3541 (3)	C8—C9	1.4125 (3)
C2—H2	0.9300	C8—H8	0.9300
C3—C4	1.4129 (3)	C9—C10	1.4388 (2)
C3—H3	0.9300	C10—C11	1.4458 (3)
C4—C5	1.4142 (3)	C11—N1	1.2911 (2)
C4—C9	1.4226 (3)	C11—H11	0.9300
C5—C6	1.3600 (4)	N1—N1^i^	1.3906 (3)
C5—H5	0.9300	O1—H1	0.978 (4)
C6—C7	1.4026 (4)		
			
O1—C1—C10	122.721 (18)	C8—C7—C6	120.54 (2)
O1—C1—C2	116.367 (18)	C8—C7—H7	119.7
C10—C1—C2	120.911 (18)	C6—C7—H7	119.7
C3—C2—C1	120.079 (19)	C7—C8—C9	121.51 (2)
C3—C2—H2	120.0	C7—C8—H8	119.2
C1—C2—H2	120.0	C9—C8—H8	119.2
C2—C3—C4	121.809 (19)	C8—C9—C4	117.502 (17)
C2—C3—H3	119.1	C8—C9—C10	123.528 (18)
C4—C3—H3	119.1	C4—C9—C10	118.951 (18)
C3—C4—C5	121.360 (19)	C1—C10—C9	119.145 (17)
C3—C4—C9	119.045 (17)	C1—C10—C11	120.359 (17)
C5—C4—C9	119.570 (19)	C9—C10—C11	120.491 (17)
C6—C5—C4	121.12 (2)	N1—C11—C10	121.408 (18)
C6—C5—H5	119.4	N1—C11—H11	119.3
C4—C5—H5	119.4	C10—C11—H11	119.3
C5—C6—C7	119.739 (19)	C11—N1—N1^i^	113.45 (2)
C5—C6—H6	120.1	C1—O1—H1	104.9 (2)
C7—C6—H6	120.1		
			
O1—C1—C2—C3	179.043 (18)	C3—C4—C9—C10	0.06 (3)
C10—C1—C2—C3	−0.64 (3)	C5—C4—C9—C10	178.266 (17)
C1—C2—C3—C4	−1.51 (3)	O1—C1—C10—C9	−177.234 (17)
C2—C3—C4—C5	−176.393 (19)	C2—C1—C10—C9	2.43 (3)
C2—C3—C4—C9	1.78 (3)	O1—C1—C10—C11	1.96 (3)
C3—C4—C5—C6	176.95 (2)	C2—C1—C10—C11	−178.377 (17)
C9—C4—C5—C6	−1.21 (3)	C8—C9—C10—C1	176.247 (18)
C4—C5—C6—C7	1.46 (4)	C4—C9—C10—C1	−2.11 (3)
C5—C6—C7—C8	−0.31 (4)	C8—C9—C10—C11	−2.94 (3)
C6—C7—C8—C9	−1.12 (4)	C4—C9—C10—C11	178.703 (16)
C7—C8—C9—C4	1.33 (3)	C1—C10—C11—N1	−1.45 (3)
C7—C8—C9—C10	−177.044 (19)	C9—C10—C11—N1	177.730 (17)
C3—C4—C9—C8	−178.391 (18)	C10—C11—N1—N1^i^	179.073 (19)
C5—C4—C9—C8	−0.19 (3)		

Symmetry code: (i) −*x*+1, −*y*+1, −*z*+1.

### Hydrogen-bond geometry (Å, º)

**Table:**

*D*—H···*A*	*D*—H	H···*A*	*D*···*A*	*D*—H···*A*
O1—H1···N1	0.98	1.67 (1)	2.5671 (3)	151 (1)
