# Supplementary figures and images for: Crystal structure of 1-((1E)-{(E)-2-[(2-hydroxy­naphthalen-1-yl)methyl­idene]hydrazin-1-yl­idene}meth­yl)naphthalen-2-ol
Source: Acta Crystallogr E Crystallogr Commun. 2015 May 28;71(Pt 6):o428. doi: 10.1107/S205698901500972X (PMC4459329; doi:10.1107/S205698901500972X)

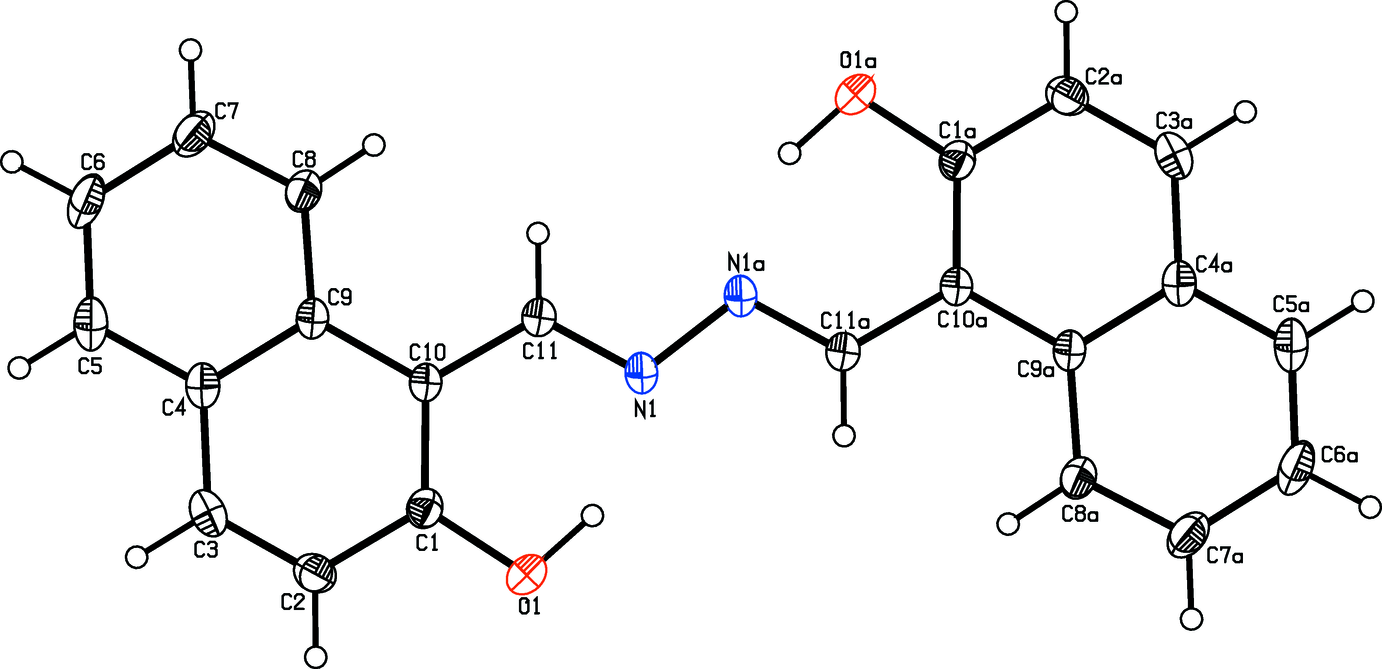

Supplement: Supplementary file 4 [file e-71-0o428-fig1.tif]
